# Removal and dispersal of biofluid films by powered medical devices: Modeling infectious agent spreading in dentistry

**DOI:** 10.1016/j.isci.2021.103344

**Published:** 2021-10-27

**Authors:** Ian Eames, Francesco D'Aiuto, Somayeh Shahreza, Yousef Javanmardi, Ramanarayanan Balachandran, Martin Hyde, Yuan-Ling Ng, Kishor Gulabivala, Sara Watson, Hywel Davies, Nicolas Szita, Janette Khajeh, Jeanie Suvan, Emad Moeendarbary

**Affiliations:** 1 Department of Mechanical Engineering, University College London, Torrington Place, London WC1E 7JE, UK; 2 Unit of Periodontology, UCL Eastman Dental Institute, University College London, London, WC1X 8LT, UK; 3 TSI, 30 Millbank, Westminster, London, SW1P 4WP, UK; 4 Unit of Endodontology, UCL Eastman Dental Institute, University College London, London, WC1X 8LT, UK; 5 Department of Biochemical Engineering, University College London, Bernard Katz Building, Gower Street, London WC1E 6BT, UK; 6 Department of Biological Engineering, Massachusetts Institute of Technology, Cambridge 02139, MA, USA

**Keywords:** Dentistry, Therapeutic procedure, Applied physics

## Abstract

Medical procedures can disperse infectious agents and spread disease. Particularly, dental procedures may pose a high risk of disease transmission as they use high-powered instruments operating within the oral cavity that may contain infectious microbiota or viruses. Here we assess the ability of powered dental devices in removing the biofluid films and identified mechanical, hydrodynamic, and aerodynamic forces as the main underlying mechanisms of removal and dispersal processes. Our results indicate that potentially infectious agents can be removed and dispersed immediately after dental instrument engagement with the adherent biofluid film, while the degree of their dispersal is rapidly depleted owing to the removal of the source and dilution by the coolant water. We found that droplets created by high-speed drill interactions typically travel ballistically, while aerosol-laden air tends to flow as a current over surfaces. Our mechanistic investigation offers plausible routes for reducing the spread of infection during invasive medical procedures.

## Highlights

• Mechanical, hydrodynamic, and aerodynamic forces drive removal/dispersal processes
• The air-rotor has the highest ability to remove and disperse infectious agents
• The aerosol cloud flows as a current and continuously settles
• Manipulating rheological properties of the fluids can suppress aerosol generation

## Graphical abstract

**Figure:**
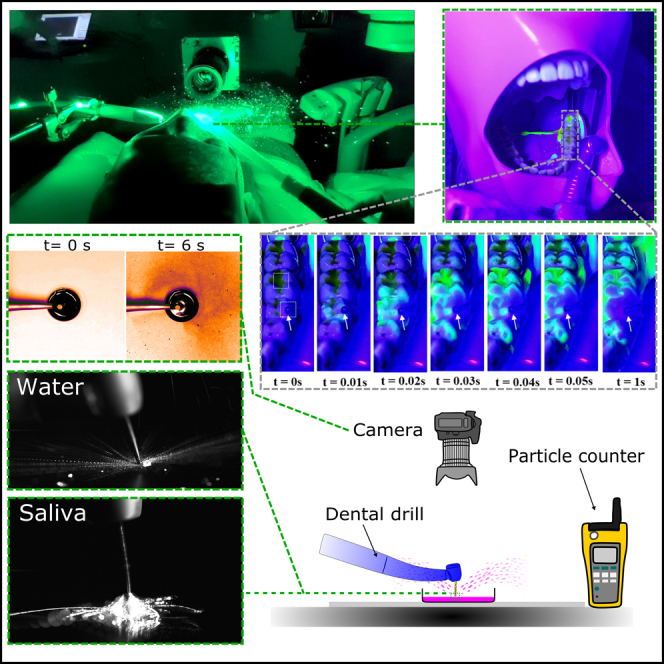

## Introduction

Medical procedures using powered instruments span a broad spectrum of specialities including orthopedics, otorhinolaryngology, ophthalmology, and dentistry, and have the potential to release biofluids into the local vicinity. Although biofluids such as blood, saliva, mucous, or tears play various roles, such as nutrient conveyance, aid digestion, and lubrication, they also have the potential to transmit viral and bacterial pathogens from one person to another (Xu et al., 2020). Several surgical procedures involve cutting bone or sinewy tissue, which demand a great deal of mechanical energy introduced either electrically or pneumatically. To mitigate tissue damage owing to the heat generated during cutting, coolant (usually water) is introduced continuously to quench the cutting surfaces. The presence of biofluids, water, air, and moving surfaces in the form of instrument tips or blades creates a potential for dispersing infectious agents including splashes, aerosols (Wells, 1934), and droplets and spreading infection through inhalation or a contact route (Tang et al., 2006).

While power-driven instrument types are common across clinical sciences, generally differing in their size and speed, dentistry represents a unique setting as it deals with the hardest tissues in the human body (i.e., enamel and dentine) requiring the fastest cutting drills and robust cooling mechanisms to prevent thermal damage to the dental pulp. Furthermore, the oral cavity, as the gateway to the body, is an open environment containing multiple biosolids and biofluids that serve as a reservoir for microbiota. The close connection with the respiratory tract and nasal pathway makes the oral environment and its associated biofluids potential reservoirs containing infectious agents that transmit diseases such as *Mycobacterium tuberculosis*, Herpes simplex, or severe acute respiratory syndrome coronavirus 2 (SARS-CoV-2).

The potential risk of spreading infection during a dental procedure involving an air-turbine drill and water coolant was recognized as early as the 1960s (Stevens, 1963). The routes, however, by which infectious agents are removed and dispersed have not been thoroughly studied (Harrel and Molinari, 2004). The current understanding on this topic, encapsulated in international guidance (PHE, 2020; WHO, 2020), is that any instrument that creates an aerosol requires specialist protocols to mitigate the risk of spreading disease. The challenge of mitigating risk partly involves characterizing what is in the air with some confusion over the definition of an aerosol. Typically an aerosol is characterized by particles whose diameter is less than 5 microns with the criterion based on the potential to be inhaled into the lower respiratory tract (Fennelly, 2020). The range for inhalation could be wider (less than 12 microns in diameter) and indeed a droplet size can shrink by as much as 80% owing to evaporation. Although the guidance employs an instrument classification based on their power to generate aerosols, they lack the underpinning fundamental science of how instruments interact with biofluid films and their potential to generate agents carrying infection (Epstein et al., 2020).

Motivated by the lack of systematic investigation on the topic (Kumbargere Nagraj et al., 2020; Volgenant and de Soet, 2018), we studied how biofluid films, that may contain viruses or bacteria, are removed and dispersed via dental instruments and procedures. We focus on the dispersal mechanism that is centered around the removal of biofluid films and crucially distinguish between coolant fluid that comes from the dental device and the potentially infectious fluid. Using imaging techniques and dyed fluid films, we analyzed the fundamental mechanisms in a laboratory setting and assessed the relevant processes under clinically relevant conditions.

## Results

### Mechanisms of aerosol and droplet generation

Droplet size has an important consequence for transport processes. It is important to clarify the terminology applied to distinguish between the different droplet sizes. The usual way to distinguish aerosols is based on the potential for deep inhalation setting a scale of 5 microns in diameter, rather than the physical processes that keep the matter in the air. Large droplets settle quickly and move ballistically, while aerosols are distinguished by their long residence time in the air. The distinction between these two groups is imprecise, especially as droplets evaporate and shrink. Based on the resolution of our probing techniques, here we distinguish between aerosols, fine droplets, and droplets corresponding approximately to <20 microns, <200 microns, and >200 microns in diameter, respectively. Application of airborne particle counter (detecting <20 μm particles), deposition (detecting >∼50 μm particles), and high-speed imaging (detecting >∼200 μm particles) techniques allowed us to probe the particles with sizes in these three categories.

**Figure 1:**
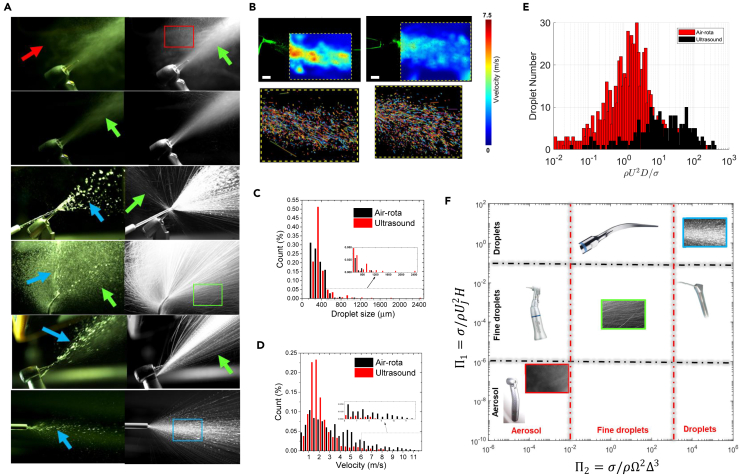
Droplet generation capacity of different dental devices (A) High-speed photography (5000 frames per s) of aerosol and droplets showing an instantaneous view (left images) and a maximum projection (right images) of 100 image sequences (corresponding to 20 ms) highlighting the trajectory of the dispersed phase. The panels (from top to bottom) correspond to air-rotor, air-rotor (without burr), low-speed drill with external 3-in-1 coolant jet and back-exhaust, ultrasonic scaler, low-speed drill, and 3-in-1. The red, green, and blue arrows show regions that aerosols (<20 microns), fine droplets (20–200 microns), and droplets (>200 microns) were generated, respectively. (B) Characterization of the spray dynamics for air-rotor (left panel) and ultrasonic scaler (right panel). The velocity contours were estimated by tracking individual droplets using PTV. Scale = 1 cm. (C and D) The distribution of droplet diameter and speed for the air-rotor and ultrasonic scaler. (E) The size and velocity of individual particles were combined to estimate the distribution of the Weber number. (F) Regime diagram showing the characterization of different dental instruments according to their potential to generate aerosols, fine droplets and droplets expressed through the movement of a liquid jet (Π_1_) or by mechanical agitation (Π_2_). The inset images are taken from the regions specified by red, green, and blue squares in (A).

We assessed three common dental devices (dental drill or air-rotor handpiece, ultrasonic scaler, 3-in-1 air-water syringe) for their potential to generate aerosols/droplets by the mechanical rotation/vibration of surfaces (bur or ultrasonic tip) or flow of air/water through small orifices (Figure 1A). Air-rotor generates the finest particles because of the fastest bur rotation and the highest airspeed. Generated droplets are propelled ballistically, while the aerosol cloud created around the drill is dispersed by the air jet and the coolant spray generates a turbulent flow of fine droplets that slows rapidly with distance owing to entrainment (Figures 1B–1D). The droplets generated by an ultrasonic scaler appear to be larger and move typically with an average velocity of ∼2 ms^−1^ (Figures 1B–1D). The air-rotor handpiece propels droplets at a greater initial velocity than the ultrasonic scaler (Figure 1B) with droplets reaching velocities of over ∼10 ms^−1^ at proximity to the rotating bur. Close to the devices, the droplets travel in a linear path while far from the device, they move with a parabolic trajectory (Figure 1B).

The ability of the instrument to convert the coolant water into droplets of different sizes depends on the balance between the surface tension force and the inertial forces created through either vibrating/rotating surfaces, air, or water flow. The different strengths in the mechanical and aero/hydrodynamic forces lead to contrasting droplet sizes with aerosols/fine droplets tending to be generated from the fast-moving surfaces (bur of the air-rotor or vibrating tip of the ultrasonic scaler). Air flow jet (expelled from the air-rotor handpiece) creates droplets, while very large droplets are formed by the water jet (3-in-1 air-water syringe). We estimated the Weber number (We), which is a measure of the relative strength of inertial to surface tension forces, to characterize the potential of the instrument to generate droplets of different sizes. The ultrasonic scaler generates larger droplets, which splash when impacting surfaces (We = ∼20), while the air-rotor creates faster moving small droplets (We = ∼1.5), that have a greater tendency to follow the airflow (Figure 1E). Based on the type and strength of the inertial forces, we categorized the ability of the instruments to convert the coolant water into aerosols, fine droplets, and droplets on a diagram in Figure 1F (see Scaling analysis in the method details section).

The inertial forces that involve in the generation of droplets from the coolant water similarly drive the removal of fluid film in the form of splashes, aerosols, and droplets of different sizes. Therefore, as examined in the next sections, aerosols and droplets can be generated from the biofluid layer through either mechanical interactions caused by a surface vibrating or rotating while in contact with the layer, aerodynamic interactions caused by the air flowing onto the layer, and hydrodynamic interactions caused by the flow of coolant water jet or droplets hitting the layer. To build up a conceptual picture of the removal processes, we designed a series of experiments starting with an idealized interaction with a fluid film and then building up to more complex interactions involving model teeth and mouth of a manikin.

### Interaction of powered instruments and adherent layers in a controlled setting

**Figure 2:**
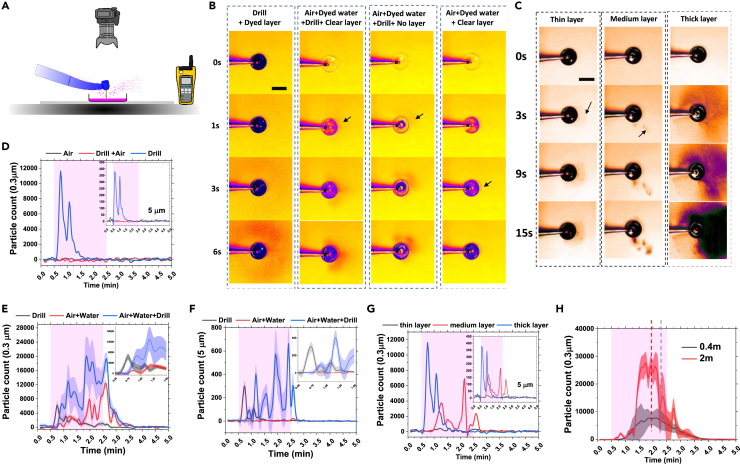
Removal and dispersal of the adherent biofluid film in a laboratory setting (A) Schematic of the laboratory setup used to analyze the mechanisms of fluid layer removal with photos taken from the above and the air sampler located 40 cm away from the dish. (B) Top view images showing the temporal evolution of dye deposition (from either dyed coolant water or dyed fluid layer) owing to air-rotor handpiece operating under different modes. Scale = 8 cm. (C) Effects of fluid layer thickness on the temporal evolution of dye deposition owing to a drill engaging with the layer. Scale = 10 cm. Arrows in (B) and (C) point to the regions that a small amount of deposition was initially detected. (D–G) Air particle count (sampled continuously over 5 min at 40 cm distance from the drill by a particle counter that measured cumulative particle count every 5 s in 0.2 L of air) as a function of time and under a variety of drill-air-water configurations. To estimate the baseline, the particle counter ran for 0.5 min prior to the operation of the drill. Then while the particle counter was continuously running, the air-rotor handpiece was operated for 2 min. After the drill operation was stopped, the particle counter was kept running for an additional 2.5 min. (D) Measurements of air particle counts (0.3 μm and 5 μm inset) when air-rotor was operated on a thick layer of water film under three operating conditions of air only (mode-2 but without the drill), drill with air only (mode-2) and drill only (mode-3). (E and F) Measurements of air particle counts (0.3 μm in (E) and 5 μm in (F)) when air-rotor was operated on a layer of water film under three operating conditions of drill only (mode-3), air with water only (mode-1 but without drill) and drill with air and water (mode-1). The insets are the zoom of the first 1.5 min. (G) Influence of the layer thickness on 0.3 μm particle count (5 μm inset) in mode-3 (drill rotation with inhibited expulsion of air and water). (H) Simultaneous measurements of particle count (0.3 μm) at 0.4 m and 2.0 m away from drill (see Figure S1F for 5 μm particles and Figures S1G and S1H for individual unaveraged curves). The curves in (D) and (G) are the smoothed data from individual measurements representing the trend. The curves in (E), (F), and (H) are the smoothened, averaged data from three independent experiments with shades indicating the standard error.

The first set of experiments involved the interaction between a fixed instrument and a fluid film focusing specifically on the air-rotor (Figure 2A), which produced the highest amount of aerosol and fine droplets with high-velocity characteristics (Figure 1E). Furthermore, examination of the air-rotor and ultrasonic scaler interacting with a thin fluid layer placed on a circular glass slide clearly suggested the negligible droplet generation by ultrasonic scaler than air-rotor particularly when operated at a fixed position (Figures S2 and S3).

To distinguish between the interconnected removal mechanisms a fixed air-rotor was operated to run the drill, water, and coolant separately and its interaction with a thin layer of biofluid under three operating modes was examined; mode-1: the normal operating condition which involves the rotation of the bur driven by the air jet while allowing the expulsion of both the air and coolant jet, mode-2: rotation of the bur driven by the air jet allowing the expulsion of the air while water expulsion was inhibited, and mode-3: rotation of the bur driven by the air jet, while both air and water jet expulsion were inhibited.

### Deposition measurements

Under these three modes and using dyed water (dyed either water film or coolant water), first we imaged the droplet deposition during continuous 2 min operation of the instrument engaging with a layer of water covering the bottom of a plastic dish (Figure 2A). Surprisingly, during the continuous operation in mode-1 and -2 no droplets and splashes were detected and only a limited number of splashes were observed immediately after operation was stopped in mode-1 (Videos S1, S2, and S3). Interestingly, in the absence of air and water jets (mode-3), upon the start of the bur rotation, the immediate interaction of the bur with the adherent fluid layer generated large splashes (Figure 2B, Video S4). This was followed by a significant deposition of dyed droplets during the 2 min interaction of the bur with the dyed layer which generated a continuous cloud of fine aerosol deposited with distinct asymmetric patterns involving a complex interaction between the turbulent airflow induced by the bur and the gravity-driven flow induced by the aerosols (Figure 2B, first column). The intensity of the dye pattern increased in time owing to the steady flow of aerosol-laden air. The aerosol-laden air appeared to flow over the edge of the dish, while the lip of the dish perturbed the low-speed aerosol-laden flow and created a narrow shadow around the dish (Figure 2B).

Video S1. Operation in mode-2 but with removed bur on dyed layer

Video S2. Operation in mode-2 on dyed layer

Video S3. Operation in full condition (mode-1) on dyed layer

Video S4. Operation of drill only (mode-3) on dyed film (∼3 mm thickness)

Next, we dyed the coolant water instead and investigate the dispersal patterns generated as the result of water jet expulsion and the interaction of the drill with water jet and the clear/undyed water film layer (Figure 2B, columns 2, 3, and 4, Videos S5, S6, and S7). When the air-rotor interacted with the clear layer under mode-1 (using dyed coolant water), significant deposition was observed (Figure 2B, second column) which was less pronounced compared with mode-3 (Figure 2B, first column). The removal of the water layer reduced the amount of deposition (Figure 2B, third column). However, a very small amount of deposition (mostly located at the proximity to the lip of the dish) was detected when bur was removed (Figure 2B, last column).

Video S5. Operation in full condition (mode-1) with the flow of dyed water on an undyed layer (∼3 mm thickness)

Video S6. Operation in full condition (mode-1) with the flow of dyed water on dish with no water layer

Video S7. Operation in mode-1 but with removed bur and flow of dyed water on an undyed layer (∼3 mm thickness)

Finally, investigation of the effects of layer thickness revealed that the intensity and the spread area of the deposition depend on the thickness of the layer when air-rotor was operating in mode-3 (Figure 2C). When the thin layer was engaged with the bur, the continuity of the dyed water layer was affected owing to the removal of the water and a limited amount of deposition was detectable (Figure 2C). However, when the thickness of the dyed layer increased a continuous reservoir of dyed water was available for removal/droplet generation and therefore the deposition area was expanded owing to a high mass flux of droplets and continuous flow of aerosol-laden current (Videos S8, S9, and S10).

Video S8. Operation of drill only (mode-3) on thin dyed water layer (less than ∼0.5 mm thickness)

Video S9. Operation of drill only (mode-3) on medium dyed water layer (∼1 mm thickness)

Video S10. Operation of drill only (mode-3) on thick dyed water layer (∼4 mm thickness)

### Aerosol measurements

Our simple photography technique was capable of capturing the dynamics of droplet (with diameters larger than ∼50 μm) deposition and suggested distinctive removal and dispersion mechanisms. However, finer droplets (typically less than ∼20 μm) are known to have a higher degree of retainment within the air and penetration into the respiratory system making them a more likely source of disease transmission. Therefore, we tested the validity of our findings for significantly finer particles by employing an airborne particle counter to probe the dispersal evolution of 0.3–10 μm droplets. Consistent with the deposition tests, no aerosolized droplets were detected under mode-2 (expulsion of air without presence of coolant water) as the flow of air pushed the dyed layer away from the bur, preventing the bur engagement with the water layer (Figure 2D). However, the presence of aerosols was recorded under mode-1 and mode-3 when the bur was engaged with a water jet or sufficiently thick fluid layer generating a significant flow of aerosolized droplets (Figures 2E and 2F).

These observations suggest that under mode (1) the aerosol (in the form of either infectious or non-infectious fine droplets) can be generated via three sources: the bur interacting with the fluid layer (Drill), the bur interacting with air/water jet that directly hitting the bur and also the dish (Air + Water + Drill), and the air/water jet direct dispersion or expulsion after hitting the dish (Air + Water). Our examination of the distinct contributions from each source to aerosol production (Figures 2E and 2F), indicated that Air + Water + Drill generated the highest levels of aerosol with a wide range of sizes (0.3–10 μm) that remained within the air beyond 2 min operation, while the Air + Water produced significantly lower aerosol levels with almost negligible amount for 5 and 10 μm droplets (Figures 2E and 2F) consistent with deposition experiments (Figure 2B). Interestingly, Drill condition produced the lowest amounts of (potentially infectious) aerosol, which diminished rapidly. Furthermore, for each condition, we observed an initial peak (occurring a few seconds after the start of the operation) with the fastest peak occurring for the Drill, suggesting the significant inertial power of fast rotating bur and effects of pre-engagement and wetting of the bur with the biofluid film. Removal of water layer from the dish had minimal effects on the production of small aerosols (0.3 μm, Figure S1D), while the amount of large aerosols (5 μm, Figure S1E) significantly increased under the presence of water layer which is consistent with the deposition measurement (Figure 2B, second and third columns).

Simultaneous measurements of aerosol at 0.4 m and 2 m distances (using two probes located on the same height) showed a lag of ∼20 s in the occurrence of the peak at the further distance (Figure 2H) while the aerosol concentration decayed after ∼60 s at both sites after the operation stopped (Figures 2H and S1F–S1H). The concentration of aerosol at the further point was dramatically lower for large droplets, with the loading of 5 and 10 μm approaching almost zero.

### High-speed imaging

**Figure 3:**
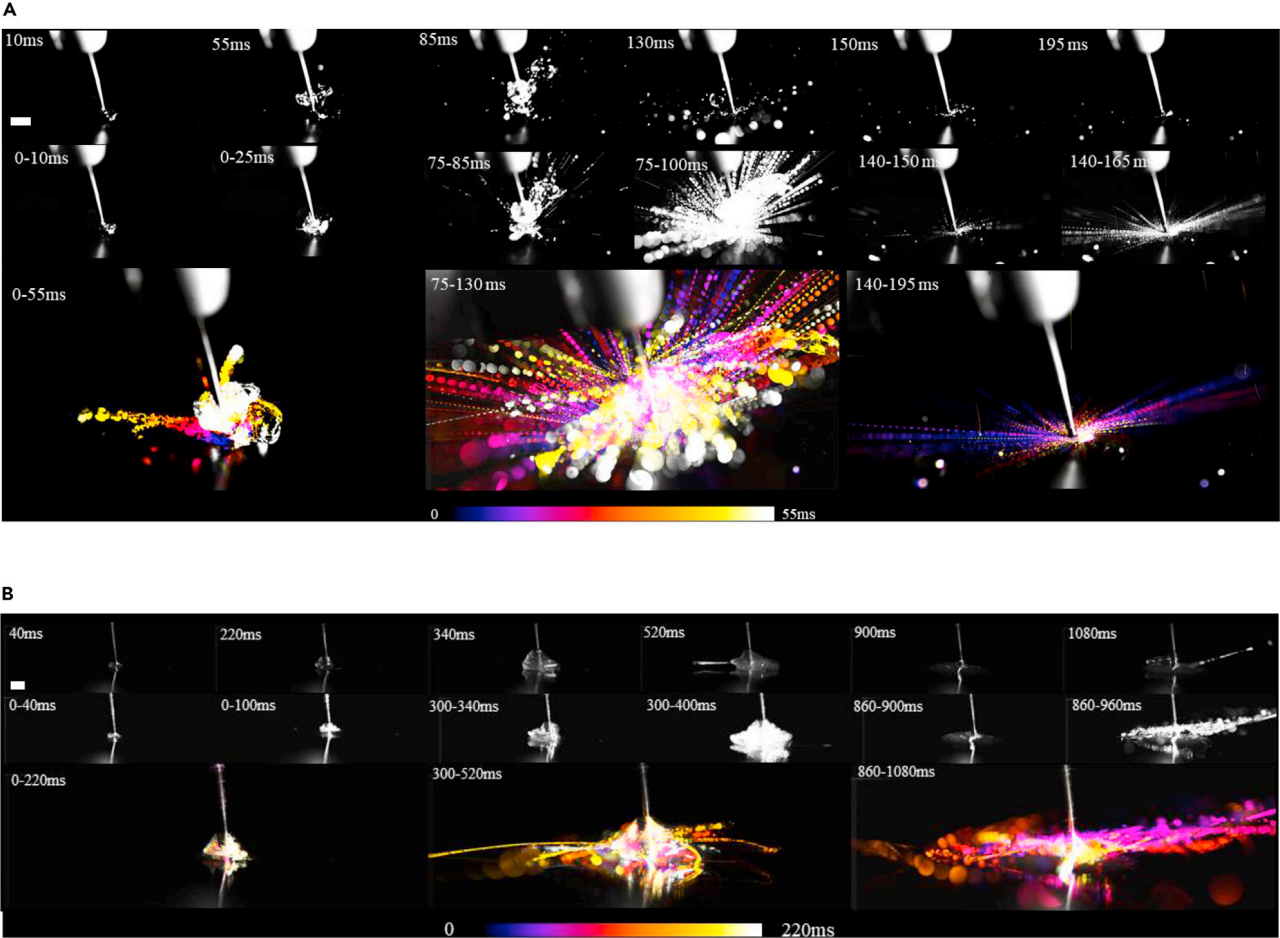
The dynamics of biofluid film removal and influence of fluid properties High-speed images (7000 frames per s) capturing the dynamics of bur rotating at ∼20,000 rad/s and engaging with water or unstimulated saliva (from a human participant) droplet/film. In (A) and (B), the top images are the instantaneous single snapshots, the middle images were created by overlaying single snapshots over a period of time. The bottom images were created by color coding single snapshots and overlaying them on top of one another. (A) The removal of a droplet of water (∼2 mm in diameter) collated in three sequences: acceleration of bur (0–0.055 s), steady full-speed rotation of bur (0.085–0.13 s), and full removal of droplet/film (0.14–0.195 s). Scale = 5 mm. (B) The interaction of a thin film of saliva (∼500 μm thickness) with bur is shown at different stages (0–0.22 s, 0.300–0.52 s, and 0.86–1.08 s) after the start of the drill rotation at t = 0. Scale = 5 mm.

Conducting high-speed imaging, we also visualized the interaction of rotating bur with the thin fluid film and investigated how the fluid film is removed and aerosolized (Figure 3). Three stages of removal were revealed: the initial rotation of the bur, steady rotation with droplet generation, and full removal of the film (Figure 3, Videos S11, S12, S13, S14, S15, and S16). During the initial rotation of the bur, the water was also rotated by the bur creating initial thin water filaments that fragmented and produced droplet ejecta whose size became progressively finer as the water layer diminished (Figure 3A). The ability of a bur to remove an adherent biofluid film depends on the rheological properties of the biofluid which was assessed by comparing the removal of water with unstimulated saliva collected from a human participant (Figure 3B). Although similar processes were observed for both water and saliva layers, the timescale of the processes was longer for saliva layer. The saliva layer initially rotated around the bur at longer timescale, longer filaments were formed and owing to increased viscosity fine droplet formation via fragmentation was greatly suppressed compared with water film (Figure 3B).

Video S11. Videos (taken with a high-speed camera) of the burr engaging with water (Videos S11–S13, Figure 3A)

Video S12. Videos (taken with a high-speed camera) of the burr engaging with water (Videos S11–S13, Figure 3A)

Video S13. Videos (taken with a high-speed camera) of the burr engaging with water (Videos S11–S13, Figure 3A)

Video S14. Unstimulated saliva collected from human participants (Videos S14–S16, Figure 3B) droplet/film

Video S15. Unstimulated saliva collected from human participants (Videos S14–S16, Figure 3B) droplet/film

Video S16. Unstimulated saliva collected from human participants (Videos S14–S16, Figure 3B) droplet/film

### Interaction of powered instruments and adherent layers assessed in a simulated clinical setting

To gain a more realistic insight relevant to the clinical situation, we next investigated the interaction between an air-rotor and teeth using either a set of adult teeth model or a manikin. Considering real tooth geometry meant that the bur and air–water flows interact with uneven/rough surfaces and the concave section of the crown (a potential saliva reservoir), while the oral cavity has a significant impact on the containment of the splashes and the aerosol flow.

**Figure 4:**
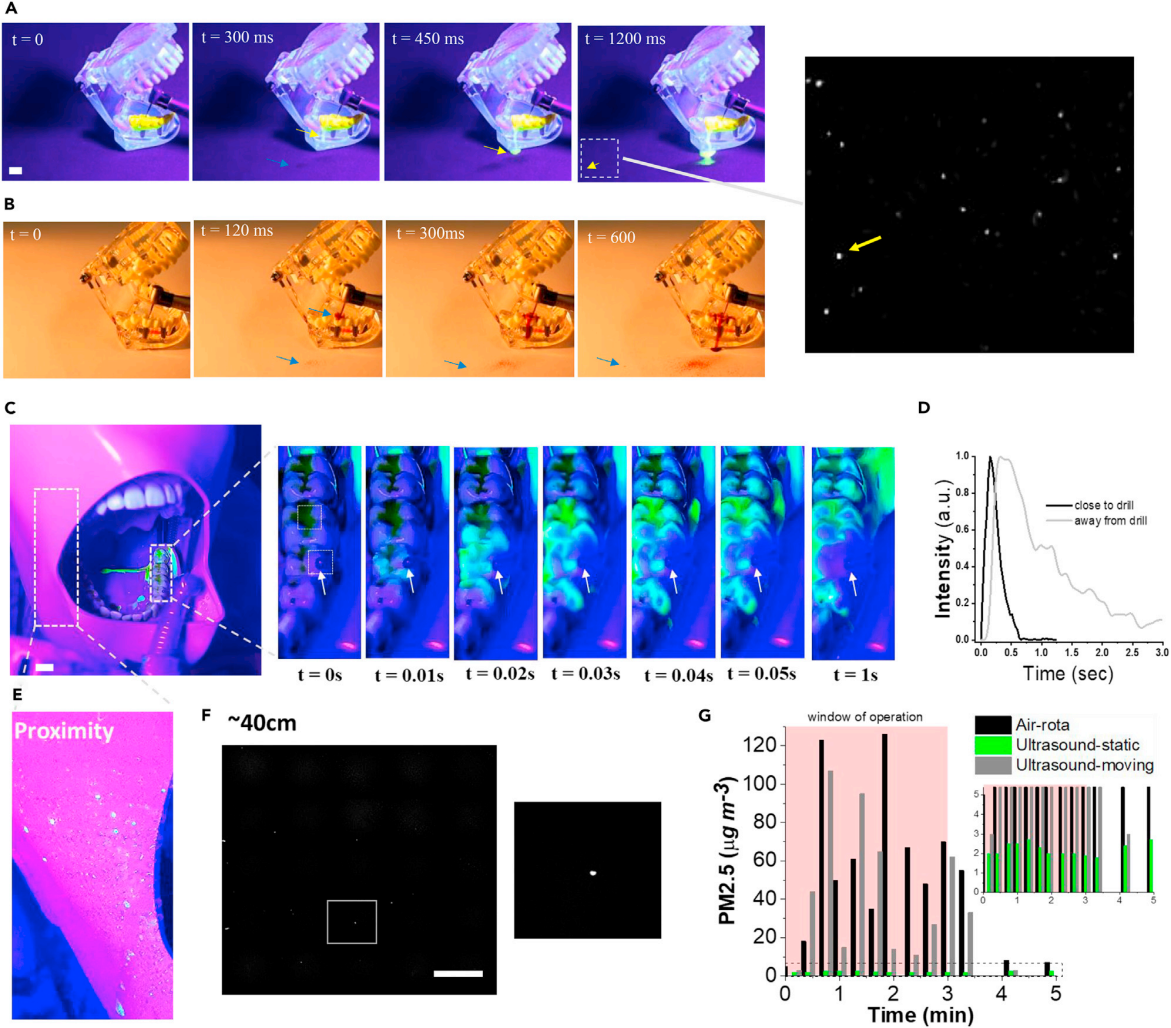
Assessment of biofluid film removal and dispersal in clinically relevant conditions (A) The removal of a fluorescently dyed simulated saliva layer by an air-rotor handpiece operating on model teeth. The teeth (45–47 ISO 3950) were coated with dye, the drill engaged with tooth 46 and the coolant water was undyed. Using a fluorescent lamp, the dyed droplet splatters were detected up to 10 cm away from the model teeth. The blue arrow points to the regions of undyed water deposition and the yellow arrows indicate the regions that dyed flow or small deposition could be detected. Scale = 1 cm. (B) The dispersal of coolant water (dyed with red food coloring) from air-rota handpiece operating on model teeth. Arrows point to the regions that a small amount of deposition could be detected. (C–G) Measurements were conducted in a simulated clinical setting with a dentist who performed procedures on a phantom head (located on a dental chair). The air-rotor handpiece pointed at the buccal cusp of the occlusal surface of teeth 35, while teeth 34–38 were coated with fluorescently dyed simulant saliva layer. (C) Image sequences show the dilution of the simulant saliva loaded with fluorescein dye. The arrows point to the tip of the bur. Scale = 1 cm. (D) Intensity profiles at two locations on the teeth (areas located close to the tip and ∼1 cm away from the tip) rapidly decayed owing to dilution by the coolant water. (E) The local splatter pattern was imaged on the surface of the manikin's face (located ∼15 cm away from a drilling point) after a 3 min continuous drilling procedure. (F) A small number of fluorescent particles (∼200 μm diameter) were detected in regions up to 0.5 m away from the drilling point. Fluorescent imaging was used to scan the tracer Petri dishes distributed up to 2 m away from the head. Scale = 1 mm. (G) Total mass loading in the air for sub 2.5-micron particles during the operation of either air-rota handpiece in a fixed position or the ultrasonic scaler operating in fixed/static or moving conditions. The inset is the zoom of the dotted area.

In the first series of tests (Figure 4A), teeth (44–47 ISO 3950) were coated with simulated saliva mixed with fluorescein dye, and an air-rotor handpiece was held by hand near the teeth with the bur contacting the occlusal surface of tooth 46 (ISO 3950). Upon turning on the air-rotor (within the first 300 ms), the presence of cooling water immediately diluted the fluorescein coating leading to the flow of dye mixture over and away from the teeth (Video S17). The convex cusps of the crown created a pool of diluted dyed water pooling at the occlusal pit. Consistent with the tests in Figure 2B, engagement of the drill with the pooled dyed water led to the generation and ejection of a small number of fine dyed droplets detected after ∼1000 ms at distances up to ∼20 cm away from the tooth (Figure 4A).

Video S17. Video of the interaction of the air-rotor (operating in mode-1) with model teeth coated with fluorescent dye The video was taken using a fluorescent lamp.

Next, the dispersion of the coolant water was examined by mixing the coolant water with the red food dye. The dyed coolant water initially coated the bur and the crown of the tooth before any observable droplet dispersion (Video S18). Within 100 ms, we observed droplet deposition generated through mechanical and aero/hydrodynamic processes. The drill's mechanical interactions (via its shank similar to Figure 1A) with coolant aerosol-jet potentially generate aerosols with the smallest droplet size (Figure 1F), while the bur engagement with the coolant pool at the occlusal pit produces fine droplets (as observed in Figure 2C). The aerosol-laden coolant jet inertially impacted the tooth surface and finer components dispersed in the air and flow as current along the surface (Videos S17 and S18).

Video S18. Video of the interaction of the air-rotor (operating in mode-1 and the coolant water dyed by a high concentration of food dye) with model teeth The video was taken using a bright field light.

In second series of tests, we used a manikin to explore the influence of drill orientation/movement and the mouth geometry in directing and confining droplet splatter. Simulant-saliva was mixed with fluorescein powder and applied to cover the teeth (34–38 ISO 3950) and the bur was engaged with teeth 35 on the buccal cusp. As soon as the air-rotor started, the simulant-saliva layer was rapidly removed from the teeth (in less than 1 s) by the water jet before the bur engaged with the tooth (Figure 4C). Although the water jet diluted the dyed simulant-saliva (as detected through an increase in the fluorescent light intensity, Figure 4D), the movement of the drill along the buccal side and its engagement with the teeth surface led to a splatter outside the mouth (Figure 4E). Droplets (mostly generated aerodynamically by the coolant jet and mechanically by the bur) were propelled through the air with a tendency to be entrained into the vortex created by flow separation at the side of the manikin's mouth, leading to the deposition of a mixture of dyed and clear large droplets on the manikin face (Figure 4E). Thorough scrutinization of all areas around the manikin indicated a discrete number of (<5) fluorescent splashes at distances up to 1 m from the head toward the foot. No dyed splashes were observed in the 4 settling plates placed within 50 cm of the manikin head, while further examination of these plates with a fluorescent microscope revealed a small number of fine spots (Figure 4F). Owing to the absence of a propelled air component, the removal pattern changed dramatically with the ultrasonic scaler as the coolant water from the agitator simply flushed the simulant-saliva layer from the teeth and splashed around the mouth only when operated continuously over several teeth.

Finally, we assessed the degree of the suspension of fine droplets (up to 2.5 microns) in the air (Figure 4G) when either air-rotor or ultrasound scaler was operated in the manikin mouth by a dentist running a 3 min routine dental procedure. We detected a rise of 120 μgm^−3^ of PM2.5 (average value 50 μgm^−3^) during the air-rotor operation which dropped dramatically when drilling ceased (10 min after the procedure was stopped, the mass loading dropped to 5 μgm^−3^). During the ultrasonic scaler operation at a *fixed position* and directed into the mouth, the air sampler did not detect a significant change showing values below 3 μgm^−3^ of PM2.5 (Figure 4G). However, when the ultrasonic scaler was swept around the mouth, and in some cases impinged on the air sampler (Figure 4F), the PM2.5 reached a maximum of 100 μgm^−3^ (average of 30 μgm^−3^). As soon as the procedure ceased, the measured particle loading in the air dropped rapidly back to the levels prior to the start of the procedure with a much faster rate than observed after ceasing the drill.

## Discussion

**Figure 5:**
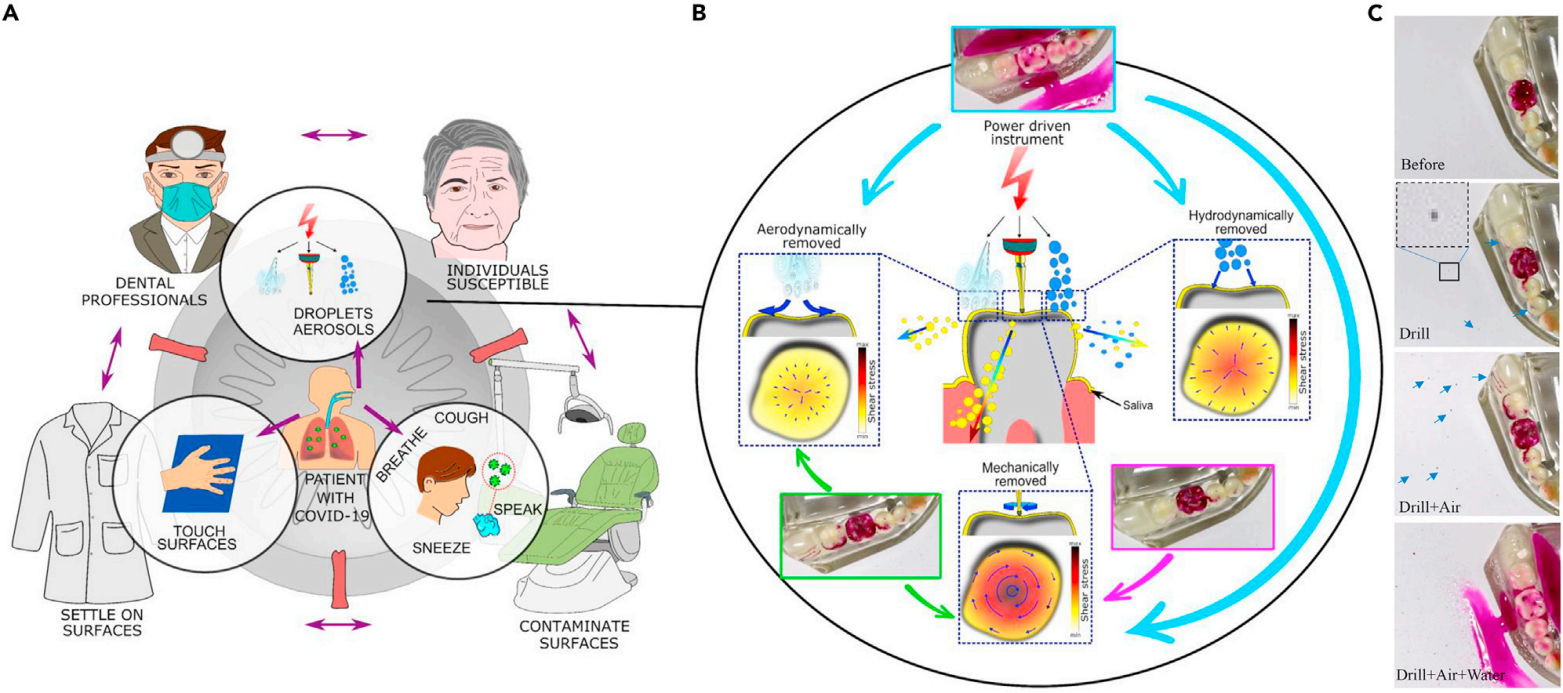
The potential risks involved in the transmission of infectious agents and the critical mechanisms for the removal and dispersal by powered instruments in dentistry (A) Schematic showing the production of aerosols and droplets by powered devices in the dental setting and their link to other transmission routes. (B) Schematic showing the removal of adherent layers through three mechanisms: mechanical (moving or vibrating surface), aerodynamic (owing to the air movement), and hydrodynamic (impact of droplets or movement of water). Color contours and arrows show the qualitative comparison between the levels of the shear stresses generated by different mechanisms. (C) The interaction between an air-rota and teeth covered with dyed simulant saliva under conditions of drill, drill + air and drill + air + water. These panels were used as insets in (B). The arrows indicate the splatter/ deposition regions.

The primary mode of disease transmission in the clinical setting is fluidic (Bourouiba, 2021), either through fine aerosols entering the air that can be inhaled, or through aerosols, fine droplets, and splashes that settle on surfaces and are transferred via contact (Peng et al., 2020; Tang et al., 2006). Infectious agents spread by medical devices affix those generated by normal pathways (Figure 5A) including breathing, speaking, coughing, and sneezing (Abkarian et al., 2020). The powered medical procedures and especially dental operations (as they use high-powered instruments) involve complex interactions between fast-moving surfaces, air, and water jets that make the assessment of the risk of the spread of infectious materials from patient to medical practitioners owing to medical procedures challenging (Figure 5A).

Numerous studies have analyzed the potential risk of disease transmission by aerosol and splatter associated with dental procedures. Splatter tests with dyed coolant water show spray deposition over large distances (Harrel et al., 1998) with a range of droplet sizes deposited within 2 m and aerosols potentially dispersed further (Allison et al., 2020). CFU microbiological assays have provided overwhelming evidence that the use of air rotors and drills enhances the spread of bacteria from the mouth compared with when transported away from the patient through breathing, speaking, or coughing (Rautemaa et al., 2006). Many previous studies do not distinguish between the clean splashes/droplets/aerosols (ejected directly from the devices (Sergis et al., 2020) or indirectly through the interaction of ejected clean flow with other surfaces) and those that contain the infectious biomaterials (mostly generated from the removal of infectious biofluid films). More recent studies have attempted to measure the distribution of aerosolized simulant-saliva laden with a virus or bacteria (such as *Streptococcus mutans*) that were continuously introduced into a phantom head mouth, while powered devices were operated on the teeth (Ionescu et al., 2020; Vernon et al., 2021). Vernon et al. (2021) analyzed the influence of mitigation strategies (such as rubber dam and aspiration) on aerosol loading and CFU on settling plates, and contrasted the spread from high-speed air rotors with lower speed electric drills. Although these quantitative measurements highlighted the importance of drill speed, air, and availability of saliva on the dispersal process (Holliday et al., 2021), the exact mechanisms by which infectious agents are removed and subsequently dispersed were not thoroughly analyzed.

The airborne spread of infection is fluidic in nature and relies on how infectious materials, that are mostly embedded within adherent biofluid films, are removed, enter the air, and are being dispersed. Therefore, assessing the risk of disease transmission from powered medical instruments first requires a thorough understanding of the removal and dispersal processes that are involved during instrument interaction with adherent biofluid films. Indeed, the small size of bacteria and especially viruses compared with a large body of fluid means that they move with the fluid and as such, tracking the fluid can provide an appropriate proxy for following transport of infectious agents. Consequently, analysis of the removal and dispersal of the adherent biofluid films using dye techniques or airborne particle counters (as in our study) can provide a valid approximation to evaluate the spatiotemporal spread of infectious agents.

Our laboratory tests identified three independent mechanisms for removing biofluid films: mechanical owing to vibrating/rotating surfaces, aerodynamic caused by airflow and hydrodynamic caused by water flow or droplet impact. Our bright-field visualizations of the deposition and aerosol measurements (Figures 2 and 4), support the view that the aerosol cloud generated during dental procedures mostly flow as a current (with an estimated velocity of ∼0.08 m/s) and continuously settles, as it moves along the surface and is not dispersed randomly unless there exists turbulence in the air (generated externally for example by ventilation systems or the movement of people).

Our data confirmed that the operation of the air-rotor has the higher ability to potentially remove and disperse the infectious agents (Figures 1, S2, and S3). As summarized in Figure 5B, three different mechanisms appear during the operation of an air-rotor with the high-speed rotation of the bur and its interaction with a film having the greatest potential for film removal and the subsequent dispersal. Depending on the geometrical constraints and operational orientation, the three mechanisms may engage with each other in an additive or subtractive fashion to remove and disperse potentially infectious biofluid film. For instance, when the bur interacts with a flat surface (Figure 2), the airflow may act to deplete the fluid film or reduce the thickness of the fluid film near the bur which decreases the amount of potentially infectious biofluid to be exposed to the drill and turn to fine aerosols through mechanical interactions. However, when the bur is located in an occlusal pit (Figure 4), the airflow may enhance the removal process and splash generation by exerting high shear forces on the film and particularly at the cusp of the teeth edge (Figure 5C). Furthermore, while we designed the experimental configurations to enhance the removal of the biofluid film coating the teeth, only a small fraction of the adherent layer was observed to be removed and deposited over a short distance (Figure 5C).

There is a growing number of practical methods to reduce the potential of infectious agents spread in dentistry, including reducing the availability of biofluids through the use of dams (Fine et al., 1993), application of suction devices to remove aerosol-laden close to the point of generation (Vernon et al., 2021). Hassandarvish et al. (2020) have shown that mouthwash can reduce viral load in biofluids in a laboratory setting. Previous measurements reported the viscosity of the human saliva to be at least twice the water viscosity (Christersson et al., 2000). Consequently, our experiments on the human saliva (Figure 3B) indicate that increasing the viscosity of a biofluid (by replacing water with human saliva) suppresses the removal. Other groups have suggested changing the rheological properties of the coolant water to reduce aerosolization (Plog et al., 2020). Therefore, manipulating the rheological properties of the fluids (biofluids and coolants) involved during a powered medical procedure is among other possible ways to suppress the aerosol generation.

In summary, our work provides a mechanistic view of the general processes of biofluid film removal and dispersal by powered medical devices, specifically in the context of dentistry. This work is an important steppingstone for proposing mitigation strategies to reduce the risk of the spread of airborne infection.

### Limitations of study

Our study focuses on modeling infectious agent spreading employing the dye technique and thus no specific microbiota or virus was used in our study. However, one limitation of such techniques is their inability to predict the levels of infectivity of the dispersed biofluid precisely. Indeed, infectious agents embedded within the fluid body may get inactivated by heat or mechanical forces generated in drilling or desiccation following droplet evaporation.

## STAR★Methods

### Key resources table

**Table:** 

REAGENT or RESOURCE	SOURCE	IDENTIFIER
**Chemicals, peptides, and recombinant proteins**		

Fluorescein sodium salt	Merck Life Science UK Limited	46970-100G-F
Rhodamine dye	Merck Life Science UK Limited	252433-1G

**Software and algorithms**		

TSI Insight 4G	TSI Inc	Version 11.2
Imaris	Bitplane	Version 7.4.2
Origin	OriginLab	2018 (b9.5.193)
ImageJ	National Institude of Health, USA	v 1.52i
Matlab	MathWorks	2017a (9.2.0.538062)

**Other**		

CMOS color camera	Photron Inc	Phantom VEO710
Temtop	Elitech	M2000
Diode laser	Coherent	Genesis MX514-1000 SLM OPS Laser-Diode System
Simulant saliva	Biotene Oralbalance	Artificial Saliva Gel
Visualizer	IPEVO	V4K Ultra HD
Air rota-turbine	NSK	Ti – Max Z 900 WL
Speed Reducing (slow) Back-exhaust	NSK	Ti-Max Z25L; Speed 1:1
Speed reducing (slow) Electric	WH	Synea WA-66 LT; speed 2:1
3 in 1	Henry Schein	1156293
Ultrasonic	Acteon	F12281

### Resource availability

#### Lead contact

Further information and requests for resources and data should be directed to the lead contact, Professor Emad Moeendarbary (e.moeendarbary@ucl.ac.uk).

#### Materials availability

This study did not generate new unique reagents.

### Experimental model and subject details

No experimental model or human subject was used in this study.

### Method details

The experimental tests were performed at the Royal National ENT and Eastman Dental Hospitals, UCLH. Two identical dental suites were used for the tests, with each serviced by clean air entry of 10 air-changes per hour (ACH) giving a potential air replenishment time of 6 minutes. Assessments in a simulated clinical setting were conducted on a phantom head with upper and lower dental arch containing 32 teeth. For the laboratory tests (performed in the UCL Environmental Fluid Mechanics Laboratory, Roberts Building), the instruments were analysed in isolation on a surface adjacent to a dental chair. The instruments used in this study are listed in Table S1.

#### Brightfield imaging

For the analysis of the sprays in Figure 1A, the instruments were held in position over a sink, illuminated with strong diffuse lighting and recorded using a high-speed CMOS camera colour camera (Phantom VEO710, Photron Inc, US). The images were recorded at a rate of 5000-7500 frames per second.

For imaging dynamics of biofluid film removal (Figure 3), approximately 100-200 μl of either water or the unstimulated saliva collected from the human participant was laid on a flat metallic surface using small pipette tip. The interaction of the drill with the water or saliva layer was investigated using high speed camera (Phantom VEO710, Photron Inc, US) with the imaging speed of 5000-7500 frames per second. The procedure of unstimulated saliva collection involves resisting the swallowing of the participant’s saliva and spitting into a small test tube every 20 s for 2 min to collect approximately 2 ml saliva.

#### Laser sheet imaging

Spray dynamics in Figure 1B was captured using laser illuminated Mie scattering technique with a laser vertical plane bisecting the dental instrument cross-section. This technique leads to capturing a much lower aerosol/droplet density in the image and is capable of measuring the velocity of individual droplets. High speed imaging of the spray was carried out using a 1000 mW, 515.3 nm continuous diode laser (Genesis MX514-1000 SLM OPS Laser-Diode System) and a high-speed CMOS camera. The camera was fitted with a 100 mm lens producing an imaging window size varying from 60 × 100 mm to 30 x 50 mm. The images were captured at a frame rate of 5000-7500 Hz. The region of interest was set to observe the near field spray characteristics. An ultrasonic scaler, high speed air-driven drill and 3-in-1 air-water syringe were used to generate sprays. TSI Insight 4G software was utilised to capture the images.

#### Fluorescein imaging

The very small size of the viruses and low diffusivity of bacteria within a body of fluid mean that a dye model is an appropriate tool for tracking the infectious biofluid transport. Furthermore, the dye technique affords high spatial resolution in terms of tracking which enables the mechanistic view of the dispersal processes to be unpicked.

To examine the mechanism of saliva removal, a series of tests were performed using a phantom head. A simulant saliva (Biotene Oral Balance) was well-mixed with sodium fluorescein salt (0.625 mg/ml in simulant-saliva), applied over the teeth and illuminated using a UV lamp. Two types of fluorescein tests were applied. In the first, dyed simulant-saliva was applied to the teeth (34-38 ISO 3950) of a manikin by a dentist and an NSK air-rotor applied to tooth 35 with the drill in contact with the buccal cusp of the occlusal surface. The drill was applied at about 30 degrees from the horizontal. In the second, dyed simulant-saliva was applied to teeth 45-47 of a full-teeth model and an air-rotor applied to the occlusal surface of teeth 46 by an experimental professor. The air-rotor was aligned 10 degrees from the vertical plane.

The fluorescein salt concentration was initially extremely high that it absorbed light when applied to the teeth (appeared as dark green), but strongly fluoresced during dilution and in the presence of UV light. To capture the potential for simulant-saliva removal, a series of petri dishes were placed at distances 20, 40 and 80 cm from the head (in the chest direction) and 40 cm above the head. All the dishes were in the same plane. Prior to each test, the lids of the dishes were removed and replaced 10 minutes after the test started. Each test consisted of 200 s continuous operation of the instruments. Photographs and videos of the experiments were recorded and analysed after the tests. During these tests, air was monitored for aerosol concentration PM2.5 and PM10 using a Temtop M2000 (Elitech) which was placed next to the phantom head. During the tests, 4 people present in the room to control the various components of the experiments.

#### Deposition tests

Splatter tests involved the interaction between powered instrument and a layer of fluid (water in Figure 2 or simulant saliva in Figures 4 and 5). The instrument was held in position and in contact with the centre of a 60 mm petri dish, placed onto a A0 sheet of white craft paper and a camera affixed above tests. Either the coolant water or the water layer was dyed using rhodamine dye (Merck Life Science, UK) and illuminated by a diffuse light source. Continuous videos were taken using a visualiser (IPEVO V4K Ultra HD) installed on top of the instrument.

#### Air sampling

During the fluorescein imaging tests within the hospital, air was sampled for a period of 10 minutes using a Temtop M2000 (Elitech). The device was placed adjacent to the phantom head and at the same level as the settling plates. The PM2.5, PM10 and particle count levels were recorded during the tests and for periods after the tests. Ventilation brought filtered air into the room and the 10 ACH for the room meant that the air born particle load was low; therefore, there is no need for the bassline subtraction and the raw data is plotted in Figure 4G.

During the splatter deposition tests in the laboratory, air was sampled using two Fluke 985 (Fluke, US) airborne particle counters that were placed flat on a workbench (pointing towards the petri dish) with a distance of 0.4 m and 2.0 m away from the dish. The ventilation system delivered unfiltered air to the laboratory (Roberts Building, UCL) and the additional components due to the local sources of aerosols was eliminated by subtracting the background concentration (Figure S1).

#### Quantification, reproducibility and image analysis

Experiments were repeated at least three times and plots and images are representative of at least three independent tests. For experiments in Figures 2E, 2F, and 2H, the curves from three independent experiments were smoothened then averaged and the standard error was calculated as indicated by the shades. Individual curves related to effects of distance is presented in Figure S1G and S1H, which show the lag time due to distance more clearly. Quantification and plotting were performed in MATLAB (Mathworks) or Origin (OriginLab). Commercially available software, Imaris (BitPlane, South Windsor, CT, USA), was used to analyse the images. After optimising image volume rendering, spot object tools were employed to automatically segment and track the droplets. An autoregressive algorithm with a maximum inter-frame distance of 150 *μ*m and a gap size of 3 *μ*m was used to calculate the position of spots over time. The droplet sizes was analysed using the “Analyse Particle” plugin in ImageJ (National Institutes of Health, USA); this technique was capable of identifying droplet size above 200 microns. The data was plotted using MATLAB.

#### Scaling analysis

The disruption of a water/air interface through mechanical agitation via a bur or vibrating tip or flow through a nozzle leads to droplets. The potential for generating aerosols (<20 *μ*m), fine droplets (20 to 200 *μ*m) or droplets (>200 *μ*m) depends on the magnitude of the forces that act on the water films. For a water jet issuing from a hole with diameter *H*, at a speed *U*_*J*_ moving through air, the inertial force of the fluid is $\rho U_{J}^{2}$. The potential for generating large droplets can be assessed by a characteristic measure based on comparing the surface tension (*σ*) that stabilises a droplet, and inertial forces, that destabilise the droplet: ${\text{Π}}_{1}=\frac{\sigma }{\rho U_{J}^{2}H}$, where *ρ* is the density of fluid. This measure is the inverse of the Weber number. When the flow is slow and inertial forces are weak, ${\text{Π}}_{1}$ is large and millimetric droplets are created. When the flow is fast and inertial forces are large compared to surface tension force, ${\text{Π}}_{1}$ is small and an aerosol will be generated. For moving surfaces with angular velocity *ω* and length scale *δ*, a centrifugal acceleration on an adherent water films scales as *ω*^2^*δ* and a nominal centrifugal force *ρω*^2^*δ*^2^ gives a second dimensionless measure $=\frac{\sigma }{\rho {\text{ω}}^{2}\delta^{3}}$. The equivalent measure for an instrument vibrating with a frequency Ω and displacement of the surface Δ is ${\text{Π}}_{2}=\frac{\sigma }{\rho {\text{Ω}}^{2}{\text{Δ}}^{3}}$. Π_1_ and Π_2_ form measures for different instruments for their potential to generate aerosols and droplets.

### Quantification and statistical analysis

Figures represent averaged or representative results of multiple independent experiments or simulations. The method details section provides details concerning the number of independent experiments. Analyses were performed using data analysis toolbox in Microsoft Excel or Origin.

## Data Availability

Additional Supplemental Items are available from Mendeley Data at https://dx.doi.org/10.17632/v9px86xh8w.1 or https://data.mendeley.com/v1/datasets/v9px86xh8w/draft?a=553d231d-9f66-46fa-b0d0-d50c3695d716.
