# Supplementary material for: Removal and dispersal of biofluid films by powered medical devices: Modeling infectious agent spreading in dentistry
Source: iScience. 2021 Oct 27;24(11):103344. doi: 10.1016/j.isci.2021.103344 (PMC8603215; doi:10.1016/j.isci.2021.103344)
Supplement: Document S1. Figures S1–S3 and Table S1 [file mmc1.pdf]

iScience, Volume *24*

Supplemental information

Removal and dispersal of biofluid films by powered medical devices: Modeling infectious agent spreading in dentistry

Ian Eames, Francesco D'Aiuto, Somayeh Shahreza, Yousef Javanmardi, Ramanarayanan Balachandran, Martin Hyde, Yuan-Ling Ng, Kishor Gulabivala, Sara Watson, Hywel Davies, Nicolas Szita, Janette Khajeh, Jeanie Suvan, and Emad Moeendarbary

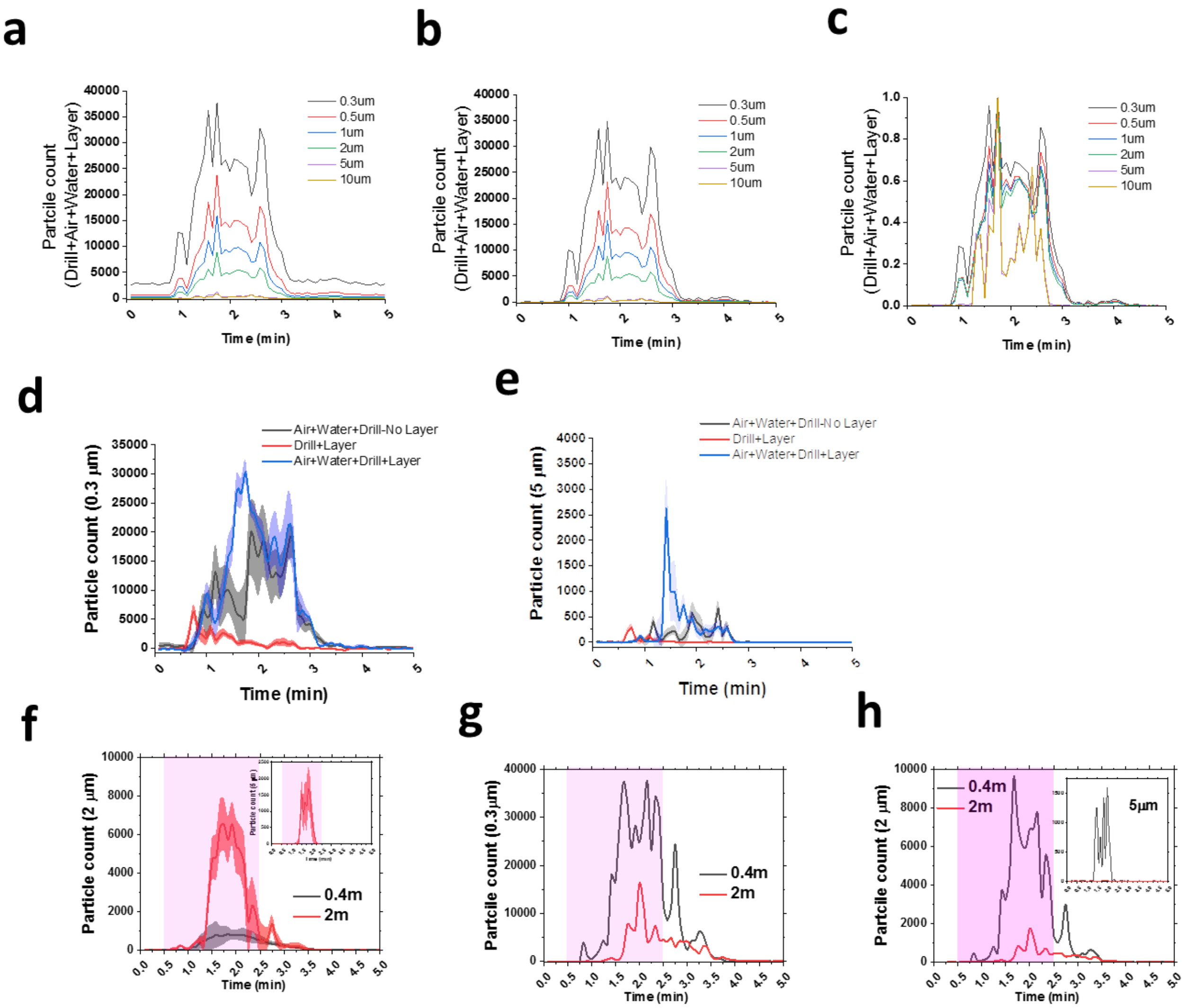

SI Figure 1: Removal and dispersion of the adherent biofluid film in laboratory setting, related to Figure 2. (a) The raw (unsmoothed) data of particle count (for particle sizes of 0.3, 0.5, 1, 2, 5 and 10 µm) as a function of time recorded by the air-particle counter for 5 min under 2 min operation of mode-1 (0.5-2.5min) **(b)** Normalisation of the curves in (a) by their baseline. **(c)** Normalization of the curves in (b) by their maximum values indicated their collapse into two distinct groups: group A: 0.3, 0.5, 1 and 2 µm and group B: 5 and 10 µm; therefore, throughout the main text we only presented curves for 0.3 µm and 5 µm as an indicative of the particle count trend in each group. **(d,e)** Effects of presence of water layer on aerosol generation (0.3 µm in (d) and 5 µm in (e)). **(f)** Simultaneous measurements of particle count (5 µm) at 0.4m and 2.0m from drill only**.** The curves in (d), (e) and (f) are the smoothened, averaged of data from three independent experiments with shades indicating the standard error. **(g,h)** Simultaneous measurements of particle count at 0.4m and 2.0m from drill. The curves are from one individual experiment.

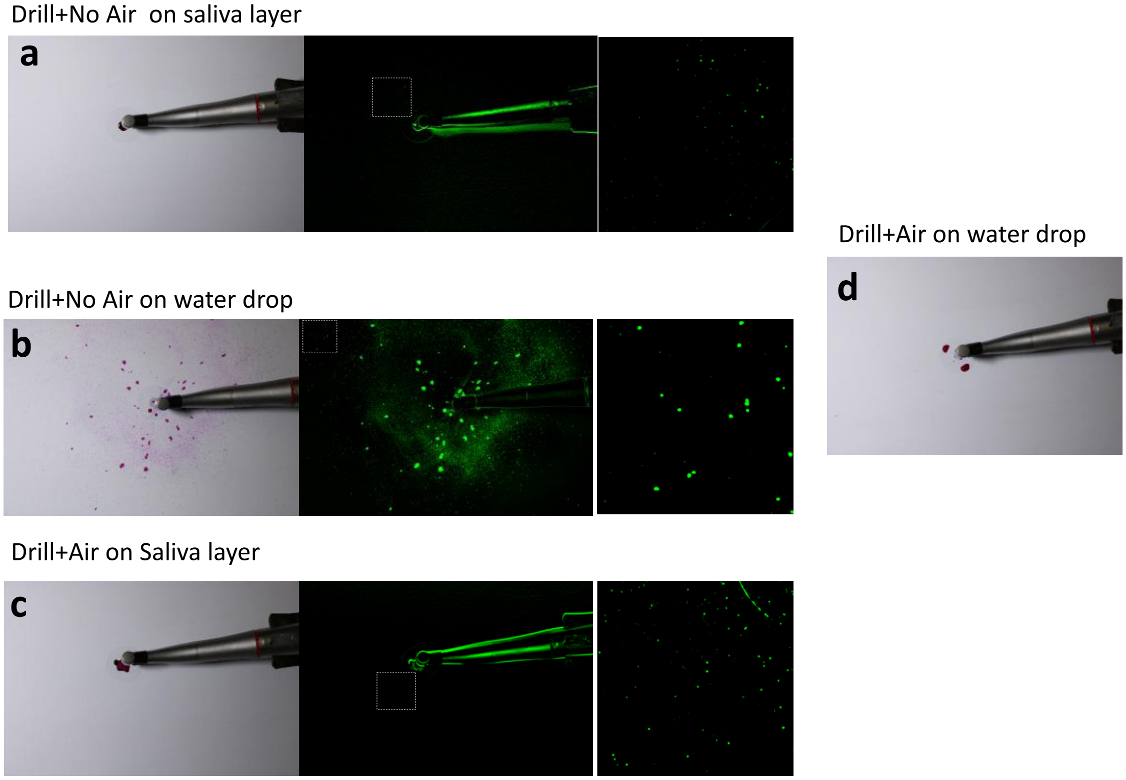

SI Figure 2: Splatter tests on a thin fluid layer placed on a circular glass slide, related to Figures 1 and 2, for **(a,b)** drill (no air) on saliva or water, respectively and **(c,d)** drill with air on saliva or water (respectively). Before and after procedure images were subtracted to generate the intensity images. The farthest areas with detectable particles are shown in the representative zoomed regions. No splatter or aerosolisation was detected in **(d)** and hence processed images were excluded.

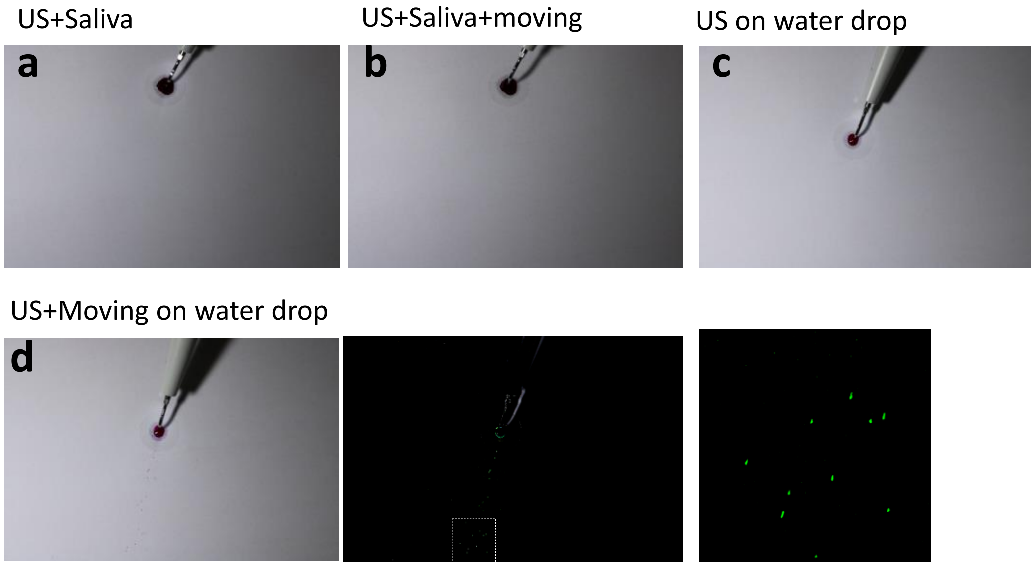

SI Figure 3: Splatter tests on a thin fluid layer by an ultrasonic (US) agitator from a circular glass slide, related to Figures 1 and 2, for **(a)** saliva and instrument fixed position, **(b)** saliva and moving instrument. **(c)** instrument fixed on water drop. **(d)** Movement of the instrument around water droplet generated splatters which was imaged and analysed using the methods explained in **SI Fig 2**.

SI Table 1: Summary of the dental instruments used in the tests along with their specifications and relevant information, related to Fig 1.

Device name	Manufacturer	Angular (Ω) velocity or vibrational frequency (f)	Air flow rate	Water flow rate	Geometry
Air rota-turbine 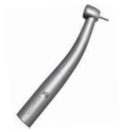	NSK (Ti – Max Z 900 WL)	350 – 380 k rpm<br><br>180-300 k rpm (cutting)	3 Bar PSI<br>43 - 53 NL/min	45 mL/min<br>: Z900WL<br>=φ0.43mm	4 chip air holes and 4 spray holes
Speed Reducing (slow)<br><br>Back-exhaust	NSK (Ti-Max Z25L), Speed 1:1	0 – 40k rpm	1.5 NL/min<br>φ0.4mm	45 mL/min<br>φ0.5mm	Z25L = 2 holes (below is water, up is chip air)
Speed reducing (slow)<br><br>Electric	WH – Synea WA-66 LT, speed 2:1	0 – 20k rpm	2 bars<br>1.5 Nl/min	50ml/min	
3 in 1 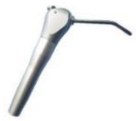	Henry Schein	0	Estimated to be 1.5NL/min	180 ml/min	1.5 mm hole
Ultrasonic 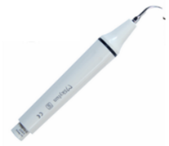	Acteon (built in)	28-36 kHz	0	7-40ml/min	Displacement is about 30 microns (Lea, Landini & Walmsley 2002)
